# Frequency-Based Maternal Electrocardiogram Attenuation for Fetal Electrocardiogram Analysis

**DOI:** 10.1007/s10439-022-02959-4

**Published:** 2022-04-11

**Authors:** Pooneh Roshanitabrizi, Anita Krishnan, Catherine Ingbar, Tyler Salvador, Anqing Zhang, Mary T. Donofrio, Rathinaswamy Govindan

**Affiliations:** 1 Sheikh Zayed Institute for Pediatric Surgical Innovation, Children’s National Hospital, 111 Michigan Ave. NW, Washington, DC 20010 USA; 2 Division of Cardiology, Children’s National Hospital, Washington, DC USA; 3 University of Arizona College of Medicine-Phoenix, Phoenix, AZ USA; 4 Biostatistics and Study Methodology, Children’s National Hospital, Washington, DC USA; 5 Prenatal Pediatrics Institute, Children’s National Hospital, Washington, DC USA

**Keywords:** Abdominal electrocardiogram, Fetal electrocardiogram, Spectral coherence, Maternal electrocardiogram

## Abstract

Fetal electrocardiogram (ECG) waveform analysis along with cardiac time intervals (CTIs) measurements are critical for the management of high-risk pregnancies. Currently, there is no system that can consistently and accurately measure fetal ECG. In this work, we present a new automatic approach to attenuate the maternal ECG in the frequency domain and enhance it with measurable CTIs. First, the coherent components between the maternal ECG and abdominal ECG were identified and subtracted from the latter in the frequency domain. The residual was then converted into the time domain using the inverse Fourier transform to yield the fetal ECG. This process was improved by averaging multiple beats. Two fetal cardiologists, blinded to the method, assessed the quality of fetal ECG based on a grading system and measured the CTIs. We evaluated the fetal ECG quality of our method and time-based methods using one synthetic dataset, one human dataset available in the public domain, and 37 clinical datasets. Among the 37 datasets analyzed, the mean average (± standard deviation) grade was 3.49 ± 1.22 for our method vs. 2.64 ± 1.26 for adaptive interference cancellation (*p*-value < 0.001), thus showing the frequency-based fetal ECG extraction was the superior method, as assessed from our clinicians’ perspectives. This method has the potential for use in clinical settings.

## Introduction

Each year, there are approximately 24,000 stillbirths in the United States^20^ and an estimated 2.6 million stillbirths worldwide.^9^ Unfortunately, these rates have not dropped since 2006.^20^ About 25-40% of these deaths are unexplained, and fetal rhythm disorders such as long QT syndrome are suspected to cause at least 3–10% percent of these unexplained deaths.^12^ Additionally, 1–3% of pregnancies experience fetal arrhythmias.^31^ Approximately 10% of the referral population of arrhythmias are life-threatening.^32^ Without early diagnosis and treatment, arrhythmias and repolarization abnormalities can progress to hydrops fetalis or death. Fetuses have high mortality after hydrops development.^29,30^ They can also cause preterm delivery or the need for cesarean section delivery which increases both infant and maternal morbidity and mortality.^30^ When appropriately detected and treated before hydrops fetalis, the prognosis for fetal arrhythmias is favorable, with up to 96% survival.^23,29,34^

Fetal monitoring is usually performed through various techniques such as cardiotocography,^5^ magnetocardiography,^14^ Doppler ultrasound,^16^ and echocardiography.^2^ However, none of these techniques can translate to a continuous, portable monitor due to the use of unwieldy Doppler probes and machines in cardiotocography and fetal echocardiography, and the demand for a costly environment shielding for magnetocardiography. Electrocardiography^1^ is the most commonly used device for detecting arrhythmias and is applicable in pediatrics due to its moderate cost and accessibility, but is not used routinely for fetal monitoring. The existing technology for fetal electrocardiogram (ECG) monitoring is primarily designed for heart rate monitoring in fetuses past 36 weeks of gestation or relies upon signal averaging.^4^ The basic framework of these existing fetal ECG devices is similar—a set of electrodes is applied to the mother and linked to a software analysis package *via* an amplifier. Approaches vary in the way the electrodes are arranged (lead montage), user interface, and signal processing algorithm. Currently, accurate, noninvasive monitoring of the fetal ECG is challenging due to the lack of direct contact with the fetus, and a very weak fetal ECG overlapped with a high amplitude maternal ECG. This is compounded by other disturbances such as power line noise, maternal muscle, respiration activity, fetal movement, and background noise.^17^

Traditional signal processing techniques have previously been proposed to separate fetal ECG from maternal ECG, including blind source separation methods (such as principal/independent component analysis (PCA/ICA)),^8,11,13,33,37–39^ template matching,^8,10,19^ and adaptive filtering.^18,21^ Blind source separation techniques require multiarray data to decompose a raw signal into independent components and extract fetal ECG. In the template matching techniques, one maternal QRS complex is considered as a template, which is searched for across the entire dataset. The matching occurrences are subtracted from the template to reduce the maternal ECG amplitude to the baseline activity. Adaptive filtering techniques use an input as the reference and another input as the primary signal. Martinek *et al.*^21^ used an adaptive filtering technique in the frequency and time domain to detect fetal ECG. This approach first regressed abdominal ECG against maternal ECG in the frequency domain. Then, the regression coefficients were used as filter coefficients to filter out the maternal ECG from the fetal ECG. Another fetal ECG technology based on adaptive filtering was established at the Johns Hopkins University/Applied Physics Laboratory [Patent numbers 6751498 (2004) and 7869863 (2011)]. Their adaptive interference cancellation (AIC) method^24^ used the standard least mean square (LMS)^35^ technique and included four components: (1) bandpass filter between 0.5 and 60 Hz, (2) canceling maternal ECG using a time-based approach, (3) enhancing the fetal ECG signal (improving the signal to noise ratio), and (4) estimating the fetal heart rate using either peak location detection using a running autocorrelation estimate or R wave detection and R–R interval measurement. Krupa *et al.*^18^ presented an adaptive noise canceler based on a neuro-fuzzy inference system to extract fetal ECG. They estimated the filter coefficients using the normalized LMS by minimizing the mean square error. Despite the efforts made by these methods proposed for fetal ECG separation, translation to the clinical setting has been slow, and the separation of fetal ECG and maternal ECG remains a major challenge.

Therefore, we developed a new frequency-based approach to separate fetal ECG from maternal ECG automatically. Additionally, we improved the signal content by averaging the fetal ECG over multiple complexes. A preliminary version of this work with 16 datasets was presented at the Pediatric Academic Society.^27^ In this paper, two clinicians measured cardiac time intervals (CTIs). We compared our method with the traditional signal processing techniques and evaluated its performance using one synthetic dataset, one real public dataset, and 37 clinical datasets.

## Materials and Methods

### Simulated Dataset

To synthetically produce a maternal-fetal ECG, we employed a publicly available model written in the MATLAB environment.^7,22^ This model approximates ECG cycles using a set of Gaussian kernel functions and produces a realistic ECG for a range of different heart rates, sampling frequencies, PQRST-complex morphologies, and noise levels. We generated the synthetic maternal-fetal ECG with the following parameters: (1) sampling frequency of 750 Hz, (2) signal length of 4 min, (3) abdominal signal (including both maternal ECG and fetal ECG) to noise ratio of 8 dB, (4) fetal to maternal signal ratio of − 3 dB, (5) maternal and fetal heart rates of 60 and 110 beats per minutes (bpm), respectively.

### Public Dataset

To assess the performance of our method using a public dataset, we selected one real dataset (subject one) from the Set A of the 2013 PhysioNet/Computing in cardiology challenge database.^28^ The dataset includes four noninvasive abdominal signals containing fetal ECG, recorded with a sampling frequency of 1 kHz for 1 min. Reference locations of R peaks were annotated based on a fetal scalp electrode.

### Clinical Dataset

In 2016, our team developed a research prototype device and began testing it under an institutional review board-approved study with patient consent at Children’s National Hospital (#Pro00007309; June 12, 2020). The device consisted of standard ECG electrodes and a Biopac MP150 data acquisition system. This device recorded 5–7 channels of maternal ECG and 8–16 channels of abdominal ECG (mixture of fetal ECG and maternal ECG). Data was acquired from singleton pregnant women at Johns Hopkins University or Children’s National Hospital Cardiology Clinic for 5 min. Deidentified datasets from Johns Hopkins University were studied under a data use agreement. Table 1 shows the data summary, including the number of subjects studied at each center, the total number of studies performed at each center, gestational age (GA), the sampling frequency for different studies, and year of data acquisition. It needs to be mentioned that the performance of our approach is not dependent on the sampling frequency of ECG. For reference purposes, the sampling frequencies are reported in Table 1.

**Table 1:** Summary of the acquired data information.

Center	# Cases	# Studies	GA (weeks)	Sampling frequency (Hz)	Year
CNH	20	34	27±7 in the range [16, 37]	250: #2; 500: #25; 2000: #7	2016–2020
JHU	26	105	32±5 in the range [24, 41]	250: #7; 500: #4; 750: #94	2002–2003

*CNH* Children’s National Hospital, *JHU* Johns Hopkins Hospital

**Figure 1:**
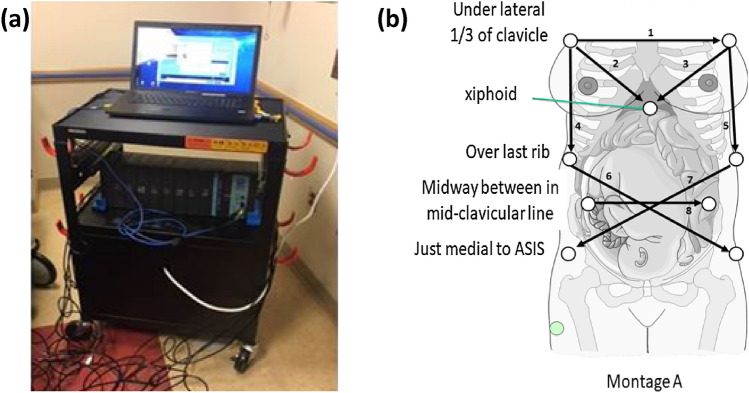
The device used for data acquisition at Children’s National Hospital; (a) system hardware and (b) lead montage.

For data collection, the position of the noninvasive ECG lead vector montage was adaptable and independent of the fetal position. Electrodes were arranged densely to avoid the loss in fetal ECG during fetal motion. In Fig. 1, one version of lead montages used in data collection is presented.

### Signal Processing Approach

**Figure 2:**
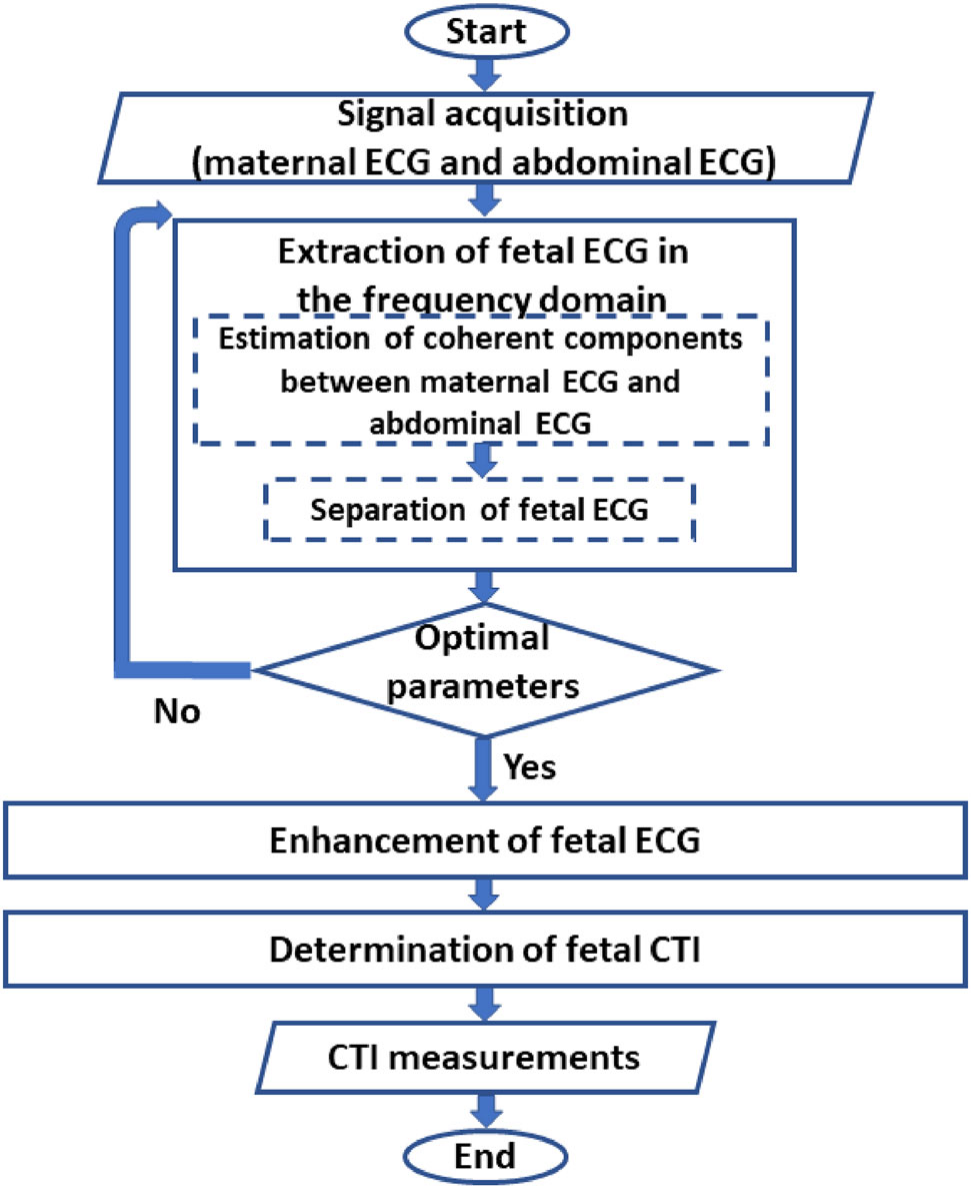
Flowchart of the method proposed for fetal ECG visualization and CTI measurement.

In Fig. 2, an overview of the proposed fetal ECG extraction method is presented including, (1) extraction of fetal ECG in the frequency domain, (2) enhancement of fetal ECG, and (3) determination of fetal CTIs. First, abdominal ECG and maternal ECG were pre-processed to remove noise and baseline wandering. Then, coherent components between maternal ECG and abdominal ECG were estimated using the null-coherence approach^15^ with the optimal parameters and subtracted from the abdominal ECG to leave the fetal ECG as residual. After that, fetal ECG was enhanced using the averaging technique^4^ for CTI measurements. These steps are described below.

#### Extraction of Fetal ECG in the Frequency Domain

Both abdominal ECG and maternal ECG were high-pass filtered (0.5 Hz cutoff) to remove baseline wandering using a fourth-order Butterworth filter on both forward and reverse directions of the signal. Then, the null-coherence approach was employed to separate the coherent components between the abdominal ECG (reference signal) and maternal ECG (source signal) to obtain fetal ECG as residual using the following steps.

#### Estimation of Coherent Components Between Maternal ECG and Abdominal ECG

In this study, we divided the data into 1-min inspection windows to attenuate the maternal ECG. Our coherence estimation followed the Welch periodogram approach.^26^ Both abdominal ECG and maternal ECG were split into 3-s independent epochs (*j*=1,…,*N*). In this paper, *N* is 20 because the 1-min inspection window consists of 20 3-s epochs. The choice of 3 s as the Fourier transform length was made to have an optimal spectral estimate while not compromising the estimate. Next, the mean value was subtracted from the data in each epoch, and the data was transferred to the frequency domain using the Fourier transform. Then, in the *j*th epoch, the periodograms of maternal ECG (${S}_{\rm mECG}^{j}$) and abdominal ECG (${S}_{\rm aECG}^{j}$) and the cross-spectrum (${S}_{\rm aECG,mECG}^{j}$) between them were calculated as follows:

1 $${S}_{\rm aECG}^{j}(\upomega )={{|F}_{\rm aECG}^{j}(\upomega )|}^{2},$$

2 $${S}_{\rm mECG}^{j}(\upomega )={{|F}_{\rm mECG}^{j}(\upomega )|}^{2},$$

3 $${S}_{\rm aECG,mECG}^{j}(\upomega )={F}_{\rm aECG}^{j}(\upomega )\times {F}_{\rm mECG}^{j\dagger}(\upomega ) ,$$

where ${F}_{\rm aECG}^{j}$ and ${F}_{\rm mECG}^{j}$ denote the Fourier transform of abdominal ECG and maternal ECG for the *j*th epoch, respectively. ω is the frequency in Hz. | . | indicates the magnitude operation. $\dagger$ represents the complex conjugate operator. Using these spectral quantities, the coherent components between abdominal ECG and maternal ECG were calculated using the spectral coherence (${\rm Coh}_{\rm aECG,mECG}(\omega )$) defined in (4). The coherence value is in the range [0, 1], where 0 and 1 show the asynchrony and synchrony, respectively, between the two signals. The confidence level of the coherence at every frequency was calculated using $1-{\left(1-\alpha \right)}^{1/(N-1)}$,^15^ where $N$ is the number of the segments involved in the spectral estimation (*N* = 20 in this study). $\alpha$ is the significance level, which was set to 0.99.^26^ Only when a significant coherence was determined between maternal ECG and abdominal ECG, the procedure was continued to attenuate the maternal ECG by (5) and (6).

4 $${\rm Coh}_{\rm aECG,mECG}\left(\omega \right)= \frac{{\left|\sum_{j=1}^{N}{S}_{\rm aECG,mECG}^{j}\left(\upomega \right)\right|}^{2}}{\left(\sum_{j=1}^{N}{S}_{\rm aECG}^{j}\left(\upomega \right)\times \sum_{j=1}^{N}{S}_{\rm mECG}^{j}\left(\upomega \right)\right)}.$$

#### Separation of Fetal ECG

To separate fetal ECG, an impulse-response transfer function (${H}_{\rm aECG,mECG}(\omega )$) was defined as follows:

5 $${H}_{\rm aECG,mECG}\left(\omega \right)=\frac{\sum_{j=1}^{N}{S}_{\rm aECG,mECG}^{j}\left(\upomega \right)}{\sum_{j=1}^{N}{S}_{\rm mECG}^{j}\left(\upomega \right)}.$$

For each 3-s epoch, maternal ECG was attenuated in abdominal ECG to leave fetal ECG as residual in (6):

6 $${F}_{\rm fECG}^{j}\left(\upomega \right)={F}_{\rm aECG}^{j}\left(\upomega \right)-\left({H}_{\rm aECG,mECG}^{\dagger}\left(\upomega \right)\right)\times \left({F}_{\rm mECG}^{j}\left(\upomega \right)\right),$$

The fetal ECG was then converted back to the time domain for enhancement.

#### Optimizing the Parameters

To identify potentially extra and missed beats, we defined the lower and upper boundaries for the fetal heart rate at 105–190 bpm.^36^ Inability to detect low amplitude signals can cause artificially low heart rate. Noisy signal, in contrast, can cause false detection of maternal ECG as a fetal beat and inaccurately high heart rate. Thus, a good quality fetal ECG should provide the minimum number of extra and missed beats.

To select the optimal parameters, we investigated the different combinations of maternal and abdominal channels for the best results. For the fetal ECG obtained for each combination, a loss function ($l$)^36^ was defined to estimate the number of missed and extra beats as follows:

7 $$l={\rm missed}+{\rm extra},$$

8 $${\rm missed}=\left(\sum \frac{{\rm RRi}}{{\rm median}\, {\rm RR}\, {\rm interval}}\right)-M,$$

9 $${\rm extra}= E-\left(\sum \frac{{\rm RRe}}{{\rm median}\, {\rm RR}\, {\rm interval}}\right) ,$$

where RRi and RRe denote an RR interval that is more than 0.5714 s and less than 0.3158 s, respectively. $M$ and $E$ represent the total number of intervals that exceed 0.5714 s and drop below 0.3158 s, respectively. Finally, the best channel combination was identified as the one that yielded the minimum loss function.

#### Enhancement of Fetal ECG

To reduce the presence of poorly attenuated maternal complex in the fetal ECG, an averaging technique was applied. First, R peaks were determined using the Pan-Tompkins method^25^ and the fetal heart rate was calculated. To ensure only genuine beats were used in the averaging, we included only the beats that yielded heart rate in the range [105–190] bpm, expected for a fetus.^36^ Finally, we improved fetal ECG quality by averaging the cardiogram using 0.5 s of data before and 0.6 s of data after each R-wave. This time duration included the QRS complexes from the previous and the following cycles.

#### Determination of Fetal CTIs

Two fetal cardiologists first evaluated the quality of the enhanced fetal ECG and graded it using a scale of 1–5,^27^ where 5 indicates a perfect signal for CTI measurements and 1 shows bad signal quality. For those signals with an average scale value greater than 4, two fetal cardiologists determined the fetal cardiac time points (P-onset, P-end, Q, R, S, T-onset, and T-end) on two different days, independently. Using those time points, the fetal CTIs (PR, QRS, RR, and QTc) were measured. Of which, QTc – a corrected QT, was calculated using the Bazett formula (${\mathrm{QT}}_{\mathrm{c}}=\mathrm{ QT}/\sqrt{\mathrm{RR}}$).^6^ Results were compared using the Wilcoxon signed-rank test at the 5% significance level. Inter- and Intra-observer reliability between the CTI measurements were calculated using intraclass coefficient (ICC) with a significance level of 5%. All analyses were performed in MATLAB using the statistical toolbox.

## Results

### Experimental Setup

We used a computer with a 4-core CPU (i5 with 3.2 GHz) to implement the method. All programs were written in the MATLAB environment. Our approach took around 2 s to attenuate the maternal ECG from a 1-min maternal ECG.

#### Simulated Dataset

**Figure 3:**
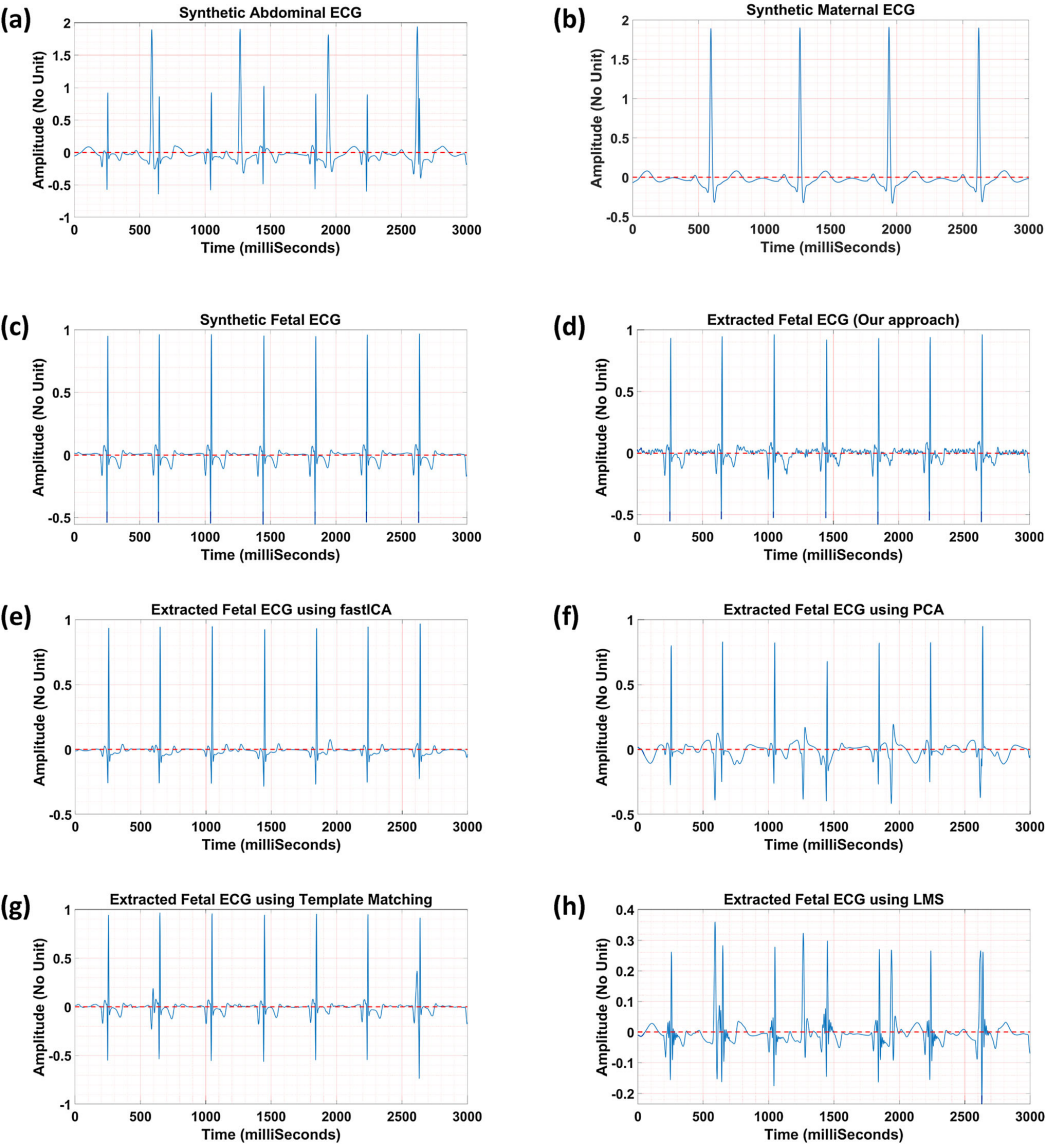
Numerical simulation to separate fetal ECG from abdominal ECG; **a** the synthetic abdominal ECG, **b** the synthetic maternal ECG, **c** the synthetic fetal ECG, the fetal ECG extracted using **d** our approach, **e** fastICA, **f** PCA, **g** template matching, and **h** LMS.

Figure 3 illustrates one sample of the fetal ECG extracted from the synthetic abdominal ECG. Figures 3a–3c show the synthetic abdominal ECG, maternal ECG, and fetal ECG (reference data), respectively. Figures 3d–3h present the fetal ECG extracted using our frequency-based method (Fig. 3d) and the open-source algorithms,^3,7^ including (1) blind source separation based on fastICA (Fig. 3e) and PCA (Fig. 3f), (2) template matching (Fig. 3g), and adaptive filtering based on LMS (Fig. 3h). Additionally, correlation values between each fetal ECG extracted and the reference data are reported in Table 2. Results show that our approach separated the fetal ECG with the highest correlation value of 98.97% when compared to the other methods. The high correlation value indicates that our method could preserve almost all the features in the fetal ECG, which did not happen to the same degree in the other methods. Blind source separation methods (PCA and fastICA) require multiple channels to separate the independent components while our method does not need this additional calculation. Adaptive filtering approaches (such as LMS) are vulnerable to maternal/fetal movement and variable heart rate. Although template matching showed a similar result to ours, selecting the right template is challenging, especially in real clinical data.

**Table 2:** The correlation coefficient between different fetal ECG results and the reference one.

	Our approach	fastICA	PCA	Template matching	LMS
Correlation coefficient	98.97%	89.63%	77.61%	96.36%	87.71%

#### Public Dataset

**Figure 4:**
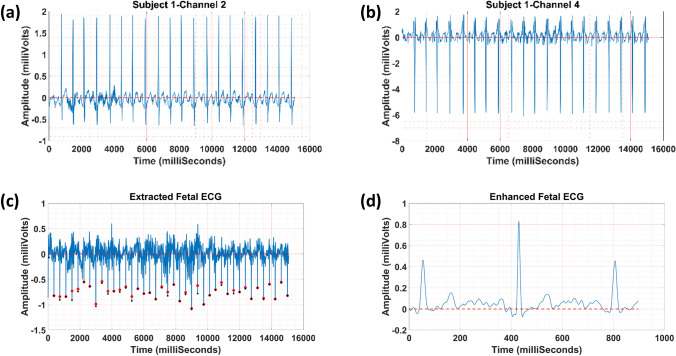
An example of fetal ECG separation from abdominal ECG using subject one in the public dataset; (**a**) channel two of ECG tracing, (**b**) channel four of ECG tracing, (**c**) the separated fetal ECG along with the reference (red ‘o’) and extracted (black ‘o’) R peak locations, and (**d**) the enhanced fetal ECG with reversed amplitude for better visualization.

In Fig. 4, fetal ECG extraction using one public dataset is presented. Figures 4a and 4b show channels two and four of ECG tracing used as the reference and source inputs in our method, respectively. Figure 4c represents the extracted fetal ECG along with the reference (red ‘o’) and extracted (black ‘o’) R peak locations. In Fig. 4d, the enhanced fetal ECG is illustrated. Results show that our method was able to separate the fetal ECG reliably. Moreover, the fetal heart rate calculated using our approach correlated well with the fetal heart rate (available *via* annotations) calculated using the fetus’s scalp electrode (see Fig. 4c). Furthermore, the visualization of fetal ECG was improved by the averaging technique, as shown in Fig. 4d).

#### Clinical Dataset

**Figure 5:**
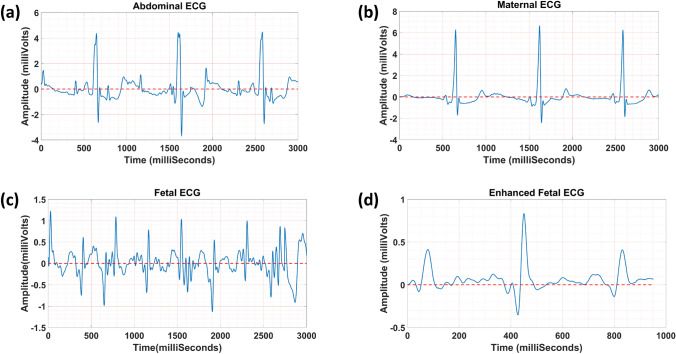
An example of clinical fetal ECG separation and enhancement; (**a**) abdominal ECG, (**b**) maternal ECG, (**c**) the obtained fetal ECG, and (**d**) the enhanced fetal ECG.

To assess the clinical results, 37 good quality datasets with three or more consecutive beats were selected. Figure 5 presents one sample of clinical fetal ECG extraction and enhancement. In Figs. 5a and 5b, abdominal ECG and maternal ECG are illustrated, respectively. Figure 5c represents the fetal ECG extracted using the null-coherence approach. Even though the amplitude of fetal ECG was almost 6 times less than the maternal ECG, our method was able to reliably attenuate the maternal ECG (see Fig. 5c). Figure 5d shows the enhanced fetal ECG, used for CTI measurements.

**Figure 6:**
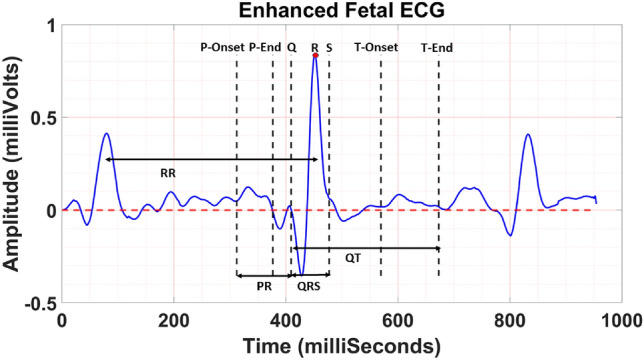
Measurement of fetal CTIs.

**Figure 7:**
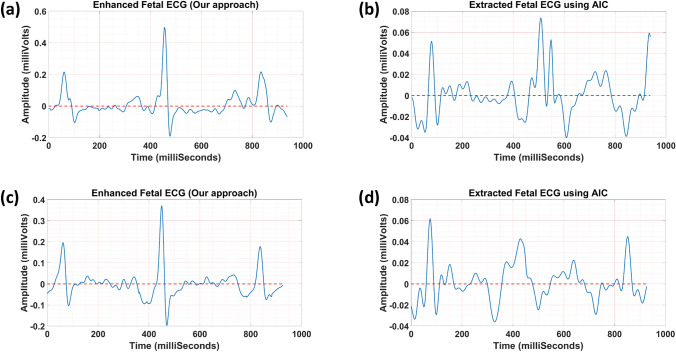
Two samples of the fetal ECG extracted using our approach (**a** and **c**) and the AIC method (**b** and **d**), used for grading and CTI measurements by clinicians.

In Fig. 6, one sample of CTI measurements is shown. Figure 7 demonstrates two samples of the fetal ECG extracted using our approach and the AIC method.^24^ In Table 3, the average grading results for our approach and the AIC method are reported. Results show that our approach yielded a higher average grade value with more reproducibility compared to the AIC method (3.49 ± 1.22 vs. 2.64 ± 1.26; Wilcoxon signed-rank *p*-value <0.001).

**Table 3:** Quantitative results obtained from clinicians’ grading.

	Our approach (average ± std^*^)	AIC (average ± std)	*p*-value
1st clinician	3.54 ± 1.3**	2.92 ± 1.43***	0.03
2nd clinician	3.43 ± 1.36**	2.4 ± 1.19***	0.002
Both clinicians	3.49 ± 1.22	2.64 ± 1.26	< 0.001

*std: standard deviation; **p-value = 0.39; ***p-value <0.001

Among the graded datasets, 48 results (including 31 results from our approach and 17 results from the AIC method; GA in range [24, 41] weeks) were graded more than 4 on average and selected for CTI measurements. Tables 4 and 5 show the ICCs with the 95% confidence interval for the measured CTIs based on our approach and the AIC method, respectively. Results show that there was a high correlation between CTI measurements made by each clinician on two different days. Also, there was a high agreement between the measured RR interval as quantified using the ICC criterion for our method and the AIC method. For other CTI measurements, there was moderate to low agreement between the two clinicians. Our method yielded more reproducibility compared to the AIC method, especially when there was no agreement for the AIC-based P- and T-wave measurements.

**Table 4:** Intraclass correlation coefficient (95% confidence interval) for the CTI measurements based on our approach.

	T	P	QRS	PR	R2R	QTc
1st Clinician	0.85 (0.72, 0.92)	0.92 (0.84, 0.96)	0.83 (0.68, 0.91)	0.95 (0.9, 0.98)	1 (0.99, 1)	0.8 (0.64, 0.9)
2nd Clinician	0.72 (0.52, 0.85)	0.59 (0.33, 0.78)	0.65 (0.42, 0.81)	0.84 (0.7, 0.92)	1 (0.99, 1)	0.6 (0.36, 0.79)
1st and 2nd clinician (1st day)	0.35 (0.11, 0.67)	0.17 (0.01, 0.71)	0.39 (0.14, 0.69)	0.34 (0.1, 0.67)	0.97 (0.94, 0.98)	0.39 (0.14, 0.69)
1st and 2nd clinician (2nd day)	0.23 (0.04, 0.67)	0.22 (0.03, 0.67)	0.27 (0.06, 0.66)	0.37 (0.13, 0.68)	0.97 (0.94, 0.99)	0.47 (0.21, 0.72)

**Table 5:** Intraclass correlation coefficient (95% confidence interval) for the CTI measurements based on the AIC method.

	T	P	QRS	PR	R2R	QTc
1st clinician	0.45 (0.13, 0.79)	0.05 (0, 1)	0.43 (0.12, 0.78)	0.37 (0.08, 0.77)	0.94 (0.86, 0.98)	0.6 (0.28, 0.84)
2nd clinician	0.51 (0.18, 0.8)	0.8 (0.55, 0.91)	0.76 (0.5, 0.9)	0.8 (0.56, 0.92)	1 (0.99, 1)	0.67 (0.36, 0.86)
1st and 2nd clinician (1st day)	0	0	0.12 (0, 0.97)	0.35 (0.06, 0.77)	0.94 (0.86, 0.98)	0.44 (0.12, 0.78)
1st and 2nd clinician (2nd day)	0	0	0.12 (0, 0.97)	0.32 (0.06, 0.77)	1 (0.99, 1)	0.37 (0.07, 0.77)

## Discussion

Early diagnosis of fetal cardiac arrhythmia is important to prevent fetal and neonatal deaths, and substantially improves the health outcome of the neonate. Electrocardiography is a safe and portable device used to diagnose arrhythmias in infants and children, but its utility for fetuses is limited by challenges, including filtering, a low signal-to-noise ratio, fetal movement, fetal orientation, amniotic fluid, and maternal characteristics. Several techniques have been proposed to separate maternal, fetal, and noise signals, including blind source separation, template matching, and adaptive filtering. However, these methods have not become a standard clinical tool. Blind source separation techniques are time-consuming, and their performance is impacted by the bandpass filtering used for the data. Template-based approaches are dependent on the maternal QRS morphology which might be changed during maternal movement and breathing. Adaptive filtering approaches are sensitive to factors such as maternal movement and fetal movement that can cause a significant baseline wandering.

In this study, we used the frequency domain, followed by an averaging technique to extract the fetal ECG from maternal ECG and enhance it to the observable CTIs. Our frequency-based approach improved the clarity of waveforms and CTIs from clinicians’ perspectives. There was low agreement between clinicians’ CTI measurements likely due to the differences in appearance from the standard pediatric ECG which required further study of normal morphology. In our next study, we plan to develop and set a rubric for grading to improve consistency. Future work is needed to increase the inter-rater reliability and to validate these methods against a gold standard. Since this study was a retrospective analysis, we were not able to alter the ECG acquisition. However, we plan to do this in the future. Our future studies also will consider a large cohort spanning the entire gestational period (21 weeks to 40 weeks) to test the robustness of our proposed approach. Additionally, although our method is fast, it is not currently suitable for real-time applications. In the future, we will optimize the processing time by converting the MATLAB codes into machine-level languages such as C/C++.
